# Pharmacological interventions for the treatment of obstructive sleep apnea syndrome

**DOI:** 10.3389/fmed.2024.1359461

**Published:** 2024-03-01

**Authors:** Jin Liu, Xiaolan Yang, Guangcai Li, Peijun Liu

**Affiliations:** ^1^Department of Central Hospital of Tujia and Miao Autonomous Prefecture, Hubei University of Medicine, Shiyan, China; ^2^Department of Pediatrics, The Central Hospital of Enshi Tujia and Miao Autonomous Prefecture, Enshi City, China; ^3^Department of Respiratory and Critical Care Medicine, The Central Hospital of Enshi Tujia and Miao Autonomous Prefecture, Enshi City, China

**Keywords:** OSAS, pharmacological treatments, respiratory disorder, ESS, CPAP

## Abstract

Obstructive Sleep Apnea Syndrome (OSAS) affects 13–33% of males and 6–9% of females globally and poses significant treatment challenges, including poor adherence to Continuous Positive Airway Pressure (CPAP) and residual excessive sleepiness (RES). This review aims to elucidate the emerging interest in pharmacological treatments for OSAS, focusing on recent advancements in this area. A thorough analysis of extensive clinical trials involving various drugs, including selective dopamine reuptake inhibitors, selective norepinephrine inhibitors, combined antimuscarinic agents, and orexin agonists, was conducted. These trials focused on ameliorating respiratory metrics and enhancing sleep quality in individuals affected by OSAS. The studied pharmacological agents showed potential in improving primary outcomes, notably the apnea-hypopnea index (AHI) and the Epworth sleepiness scale (ESS). These improvements suggest enhanced sleep quality and symptom management in OSAS patients. With a deeper understanding of OSAS, pharmacological interventions are emerging as a promising direction for its effective management. This review provides a comprehensive overview of the current state of drug research in OSAS, highlighting the potential of these treatments in addressing the disorder’s complex challenges.

## 1 Introduction

Obstructive Sleep Apnea Syndrome (OSAS) is a prevalent sleep disorder affecting approximately 9–38% of adults worldwide. It is more common in males (13–33%) than females (6–19%), with a higher prevalence among older adults. Excessive daytime sleepiness seems to have a greater impact on younger adults than the elderly. Genetic factors also contribute to the development of OSAS, emphasizing its importance as a global public health issue (1–3).

The hallmark of OSAS revolves around upper airway obstructions or dysfunctions, leading to symptoms such as intermittent hypoxemia, oxidative stress, sleep fragmentation, and excessive daytime sleepiness (4, 5). Patients with moderate to severe OSAS often encounter an array of health concerns, including cognitive impairments, and psychological ailments (4, 6–8). More alarmingly, untreated OSAS is associated with severe health issues like cardiovascular diseases, diabetes, and hyperlipidemia. Such comorbidities often worsen the prognosis for OSAS patients, affecting their quality of life and treatment adherence (5, 9–11).

Continuous positive airway pressure (CPAP) is recognized as the primary therapeutic intervention for patients with moderate to severe OSAS and is judiciously prescribed for select mild cases. While CPAP has been demonstrated to substantially mitigate symptoms such as hypoxemia, fatigue, depression, and anxiety, and diminish the associated cardiovascular mortality risk, its implementation does present certain challenges (12). However, the long-term adherence to CPAP remains suboptimal. This, coupled with the chronic nature of OSAS treatment, results in many unsuccessful treatment endeavors. Other alternatives like mandibular advancement devices and surgical interventions, though beneficial to some extent, are constrained by their cost and uncertain outcomes (13). Other non-surgical methods, such as positional therapy and transcutaneous electrical stimulation, are yet to be validated in extensive clinical trials. A profound understanding of OSAS and the exploration of new treatment modalities is crucial. This study aims to delve deeper into the pathophysiological mechanisms of OSAS, hoping to pave the way for the development of novel, safe, and effective pharmaceuticals.

## 2 Pathophysiological mechanisms and risk factors of OSAS

### 2.1 Introduction to OSAS etiology

Recent research on OSAS has enhanced our understanding that its etiology may be associated with impaired function of the upper airway dilator muscles during sleep, constricted airway passages, and reduced respiratory stability (14). This understanding opens avenues for tailored treatments based on individual patient phenotypes.

### 2.2 Inducing factors

These insights guide ongoing and forthcoming research toward developing targeted treatments tailored to diverse patient phenotypes. OSAS is a chronic disorder, originating from various anatomical abnormalities. This review concisely summarizes its pathophysiological underpinnings, encompassing both the initiating factors and risk components (4, 14). Several inducing factors contribute to OSAS, such as reduced surface tension, upper airway luminal narrowing, diminished lung volume, unstable respiratory control, dysfunctional upper airway muscles, and a low arousal threshold. Generally, pharyngeal collapse, primarily due to anatomical alterations in the upper airway, is considered a predominant etiological factor (15).

### 2.3 The role of high loop gain

The term “high loop gain,” a feedback control system in the human body, reflects the balance between ventilation and its disturbances, and its role in OSAS is associated with upper airway muscle activity and carbon dioxide concentration (14, 16).

### 2.4 Arousal threshold

The arousal threshold plays a pivotal role in the pathogenesis of OSAS. Some studies have indicated that arousals can be triggered once inhalation reaches a certain intensity (17) which subsequently impacts respiratory stability. Frequent arousals can lead to blood oxygen disturbances, resulting in fragmented sleep patterns that prevent patients from entering deeper stages of sleep, thereby exacerbating their OSAS (18–21).

### 2.5 Upper airway muscle dysfunction

This dysfunction is central to OSAS development. While effective in countering inhalation-induced negative pressure when awake, during sleep, these muscles’ activity diminishes, predisposing the pharynx to collapse and result in respiratory obstruction (22). The genioglossus muscle, a principal part of the pharyngeal dilator muscles, exhibits varied activity across different sleep stages; it’s more active during inhalation than exhalation. Therefore, the loss of reflexes between central input neurons and upper airway muscles might contribute to the onset of OSAS (22–25). Additionally, neurogenic damage or excessive muscle fatigue leading to reduced genioglossus muscle function, or pharyngeal sensory impairments, can cause airway collapse (26–28).

### 2.6 Specific risk factors

Some factors, such as a low arousal threshold, reduced lung volume, or unstable respiration, might seem normal but can still lead to OSAS (29). Smoking, which relaxes the respiratory muscles causes upper airway obstruction (30, 31). Aging, characterized by a reduction in pulmonary elastic recoil, can lead to airway collapse. Alcohol intake impacts not just sleep quality but also significantly elevates the risk for OSAS. Studies have demonstrated that elevated alcohol consumption raises the risk of sleep apnea by 25% (relative risk 1.25, with a 95% confidence interval of 1.13–1.38, *p* < 0.0001) (32). This link between high alcohol intake and increased sleep apnea risk underscores the importance of reducing alcohol consumption as a potential therapeutic and preventive approach to this condition. Additionally, gender and body composition play a role: males inherently have longer airways than females, increasing their susceptibility to passive airway collapse, while obesity may result in fat deposition within the tongue, potentially compromising the function of the genioglossus muscle and impacting lung volume. Furthermore, conditions such as menopause and adenotonsillar hypertrophy (4) are also recognized as potential risk factors for OSAS (33–35).

### 2.7 The PALM model

Eckert et al. proposed a comprehensive model in 2013 to elucidate the pathophysiological mechanisms underlying OSAS. This model incorporates Pharyngeal critical pressure (P), a Low arousal threshold (A), a High loop gain (L), and the Muscle responsiveness of the upper airway (M). The prominence of the PALM-2 phenotype allows for tailored treatment strategies aligned with the distinct pathophysiological characteristics of individual patients (36).

### 2.8 Identifying subtypes of OSAS

Obstructive Sleep Apnea Syndrome is characterized by significant health risks and symptom variability, making its management complex. Subtype analysis has been key in identifying OSA subgroups like “excessively sleepy” and “disturbed sleep,” which differ in cardiovascular risk and treatment efficacy (37). Labarca et al. (38) highlight that patients with the “excessively sleepy” subtype face a higher cardiovascular mortality risk after 5 years, emphasizing the need for customized treatment approaches (38). Specifically, the “excessively sleepy” group may require more intensive treatments to lower cardiovascular risk, while those with “disturbed sleep” could benefit from cognitive behavioral therapy focused on specific symptoms.

## 3 Diagnosis

### 3.1 Polysomnography (PSG) in OSAS diagnosis

Polysomnography is the gold standard for diagnosing OSAS. It involves the monitoring of a multitude of physiological parameters to evaluate the patient’s condition: Electroencephalography of bilateral frontal, central, and occipital lobes to assess cerebral electrical activity. Surface chin and leg electromyogram to evaluate the genioglossus muscle beneath the chin, crucial for identifying rapid eye movement sleep. Left and right eye electrooculograms to monitor eye movements. Nasal pressure sensors to measure variations in nasal airflow. Pulse oximetry for measuring hemoglobin saturation levels. Monitoring the intensity and frequency of snoring and utilizing respiratory inductive plethysmography belts, among others.

### 3.2 Home sleep apnea testing (HSAT)

In cases where patients lack severe comorbidities—like stroke, central sleep disorders, or a history of prolonged opioid use—Home Sleep Apnea Testing can be an alternative. The test should span over more than one night, as multiple nights’ records can enhance diagnostic accuracy and prognostic value. HSAT offers better patient compliance and cost-effectiveness compared to PSG. However, it falls short in sensitivity relative to PSG. Hence, if HSAT doesn’t confirm the diagnosis, PSG remains the preferred choice. Future advancements should focus on refining HSAT and improving its associated technology (39).

## 4 Pharmacological interventions in OSAS treatment

Exploring the pathophysiological complexities of OSAS paves the way for pinpointing appropriate therapeutic agents for alleviating symptoms. A range of medications, rigorously tested in clinical trials, has emerged as notably effective. Among them are drugs modulating neurotransmitters, especially impacting dopamine (DA) and norepinephrine (NA) receptor levels. This category includes selective dopamine reuptake inhibitors, norepinephrine inhibitors, combined adrenergic-anticholinergic agents, and orexin agonists. Several drugs, including modafinil, pitolisant, and solriamfetol, enhance alertness in patients, helping to alleviate daytime drowsiness and cognitive lapses. At the same time, medications such as atomoxetine, oxybutynin, reboxetine, and oxybutynin target specific muscles in the upper respiratory tract, such as the genioglossus, elevator, and tensor palatine muscles, to mitigate sleep-related breathing problems. In essence, our deepening grasp of pathophysiology informs these therapeutic directions. The effectiveness of these drugs in trials underscores the merit of this strategy, hinting at even more refined OSAS interventions on the horizon (40, 41). The relevant studies on pharmacological treatments for OSAS are summarized in Table 1.

**TABLE 1:** Summary of pharmacological treatments for OSAS.

Drug name	References	Research design	Study duration	Treatment measure		Outcome
				Treatment group (N)	Control group (N)	
Modafinil	(49)	RCT	2 weeks	200 mg/day (16)	Placebo (15)	Modafinil reduced daytime sleepiness in men with mild to moderate OSA
Modaifinil	(52)	OCT	12 months	200–400 mg/day (266)		Modafinil boosts alertness and functioning, leading to better quality of life with long-term tolerability
Modaifinil	(54)	RCT	12 weeks	200 mg/day (104) 400 mg/day (101)	Placebo (104)	Modafinil improves clinical metrics and performance with good tolerability
Modaifinil	(55)	OCT	12 weeks	200–400 mg/day (58)	Placebo (67)	Modaifinil reduced daytime sleepiness and improved sleep function and quality of life
Modafinil	(56)	RCT	4 weeks	200–400 mg/day (77)	Placebo (80)	Modafinil reduced daytime sleepiness and enhanced psychomotor vigilance
Armodafinil	(58)	RCT	2 weeks	150 mg/day (35)	Placebo (34)	Armodafinil improved driving safety and quality of life for patients awaiting CPAP therapy
Armodafinil	(50)	RCT	12 weeks	200 mg/day (125)	Placebo (124)	Armodafinil improved ESS and CGI-C scores, with MWT slightly higher
Armodafinil	(53)	RCT	12 weeks	150 mg/day (131) 250 mg/day (131)	Placebo (130)	Armodafinil mitigates the effects of excessive sleepiness on daily life
Pitolisant	(63)	RCT	12 weeks	20 mg/days (200)	Placebo (68)	In patients not using CPAP, Pitolisant enhanced daytime alertness and overall clinical status
Pitolisant	(65)	RCT	12 weeks	20 mg/day (183)	Placebo (61)	Pitolisant notably reduced sleepiness symptoms and improved patients’ condition severity
Solriamfetol	(73)	RCT	12 weeks	37.5–300 mg/day (355)	Placebo (119)	Solriamfetol enhanced patients’ alertness with favorable results
Solriamfetol	(72)	RCT	6 weeks	75–300 mg/day (62)	Placebo (62)	Solriamfetol enhanced alertness and reduced subjective sleepiness
Solriamfetol	(75)	RCT	2 weeks	150–300 mg/day (17)	Placebo (17)	Solriamfetol enhanced driving performance in those with EDS
Solriamfetol	(80)	RCT	12 weeks	37.5–300 mg/day (355)	Placebo (119)	Armodafinil enhanced driving performance and life quality in OSA patients awaiting CPAP treatment
Solriamfetol	(78)	RCT	12 weeks	37.5–300 mg/day (345)	Placebo (114)	Solriamfetol reduced daytime sleepiness
Reb-Oxy	(89)	RCT	2 weeks	Reb 4 mg-Oxy 5 mg/day (9)	Placebo (9)	Reb-Oxyreduced daytime sleepiness
AD109	(92)	RCT	3 night	Low-dose (37.5 + 2.5 mg, 32) high dose (75+2.5 mg, 25)	Placebo (28) Placebo (15)	AD109 boosted sleep quality in mild to moderate patients
Ato-Oxy	(95)	RCT	3 night	Ato 80 mg–Oxy 5 mg (32)	Placebo (20)	Ato-Oxy enhanced sleep quality in mild to moderate patients
Ato-Oxy	(96)	RCT	1 month	Ato 80 mg (15) Oxy 15 mg (10)	Placebo (10)	Ato-Oxy improved sleep with high tolerability

RCT, randomized controlled trial; OCT, openly clinical-trials; OSA, obstructive sleep Apnea; ES, excessive sleepiness; CGI-C, clinical global impression of change; CPAP, continuous positive airway pressure; Reb, reboxetine; AD109, armodafinil dimesylate; Ato, atomoxetine; Oxy, oxybutynin.

### 4.1 Modafinil and armodafinil: wake-promoting agents

Modafinil was originally developed for the treatment of narcolepsy. Its pharmacological actions have been partially elucidated, suggesting that its efficacy might be achieved through a combination of actions on norepinephrine α1, dopamine, and orexin receptors. Animal studies have further implied its association with α1B adrenergic receptors or in conjunction with cAMP response element-binding protein and mitogen-activated protein kinase promoting binding with D1 receptors in mouse wakefulness regions (42, 43). Furthermore, in rodent studies, MOD increased dopamine levels in the brain and decreased GABA release, leading to the continuous activation of brain regions associated with alertness (44, 45). Distinctively, MOD’s neuron activation is more localized in certain brain regions compared to traditional stimulants like amphetamines (46).

Interestingly, MOD is also considered a catecholamine reuptake inhibitor. Animal investigations further suggest that it can affect dopamine and norepinephrine transporters in the brains of adult monkeys, elevating the levels of DA and NA, and thereby aiding in maintaining an alert state (47). In 2004, the US FDA approved MOD as an effective treatment for OSAS, particularly beneficial for patients who still exhibit excessive daytime sleepiness (EDS) after CPAP treatment. The recommended initial dose should not exceed 200 mg (48–50). Numerous randomized controlled trials (RCT) have attested to MOD’s superior efficacy in treating OSA (51–60). An RCT conducted by Julia et al. revealed that treatment with 200 mg of modafinil for over 2 weeks significantly improved symptoms of EDS in patients with mild to moderate OSAS (52). The Epworth Sleepiness Scale (ESS) scores decreased by 3.6 points. Interestingly, the therapeutic effects were comparable, if not superior, to standard CPAP treatment. Moreover, patients expressed a preference for pharmacological intervention over CPAP. Similarly, a cross-over RCT by David and colleagues demonstrated that modafinil (400 mg for 3 weeks) ameliorated electroencephalographic activities during periods when CPAP treatment was halted (51). The study also noted enhancements in cognitive and neurobehavioral functions. A 1-year open-label study echoed the safety and efficacy outcomes observed in shorter trials, highlighting improvements in the patient’s quality of life (55).

Armodafinil, recognized as the R-enantiomer of modafinil, operates via a similar mechanism. Several RCTs have documented its beneficial effects (61). A meta-analysis by Julia and Liora incorporated 10 studies with 1466 patients (48). Their findings solidified the therapeutic value of modafinil and armodafinil. Compared to placebo, the ESS scores of patients improved by 2.2 points, and the Maintenance of Wakefulness Test (MWT) duration increased by 3 min. Clinically, symptom improvement was 1.6 times higher than that of placebo. However, some research indicates that armodafinil doesn’t alter frontal cortex MRI readings, suggesting that patients might still experience persistent hypersomnia (62).

Regarding side effects, modafinil’s most common adverse reaction is headaches, which are experienced by over 35% of users. Other mild side effects, such as nausea, diarrhea, altered taste sensations, anxiety, and insomnia, have been reported but are less prevalent. Notably, headaches remain the predominant complaint among patients (47).

### 4.2 Pitolisant: a selective H3 receptor antagonist

Pitolisant stands as a selective H3 receptor antagonist or inverse agonist. By uniquely binding to the H3 receptors, it fosters a heightened release of histamine, thereby promoting wakefulness in patients. Current studies suggest its applicability in treating conditions like narcolepsy, Parkinson’s disease, and daytime hypersomnia in patients with OSAS (63). Furthermore, pitolisant augments the levels of other monoaminergic neurotransmitters, such as NA, acetylcholine, and DA, offering additional symptomatic relief (64). The drug exhibits a plasma half-life between 10 and 12 h. It is predominantly absorbed in the gastrointestinal tract, metabolized in the liver, and excreted via the kidneys. As such, caution is advised for patients with renal insufficiency (50, 65). Dauvilliers et al. (66) conducted a study with 268 patients, of whom 75% were male. The average age of the participants was 52, and their baseline characteristics included an AHI index of 49/h and a baseline ESS score of 15.7. This study aimed to evaluate the efficacy and safety profile of Pitolisant in OSAS patients. The outcomes indicated a significant reduction in the ESS score compared to a placebo group, with no noted withdrawal symptoms post-discontinuation. Moreover, several double-blind RCTs revealed Pitolisant’s ability to alleviate fatigue and ameliorate the severity of the disorder (67, 68).

A meta-analysis involving 678 patients, including 166 with narcolepsy and 512 with OSAS, indicated that Pitolisant exhibited a significantly greater therapeutic efficacy in OSAS patients compared to those with narcolepsy. This suggests that pitolisant may be more suitable for the treatment of OSAS (69). Common adverse reactions post-pitolisant administration include headaches, nausea, insomnia, and anxiety. Notably, the drug may induce a QT interval prolongation, warranting avoidance of concurrent administration with Class 1A and 3 antiarrhythmic drugs. The use of Pitolisant is contraindicated for individuals with severe hepatic or renal impairments (50).

### 4.3 Solriamfetol: a selective dopamine and norepinephrine reuptake inhibitor

In 2019, both the European Medicines Agency and the U.S. FDA endorsed the approval of Solriamfetol (Sunosi) for the treatment of narcolepsy and OSAS in adults. Sunosi functions as a selective reuptake inhibitor for dopamine and norepinephrine within the cells, targeting their transporters to suppress reuptake (70, 71). These neurotransmitters play pivotal roles in sleep modulation and excessive daytime sleepiness (72). For patients with OSAS, the recommended initial dosage is 37.5 mg/day. Depending on its efficacy and tolerability, the dosage may be adjusted up to a maximum recommended daily dose of 150 mg (71). However, several clinical trials have tested doses as high as 300 mg without significant adverse reactions (73–76). A meta-analysis conducted by Subedi et al. revealed no significant difference in outcomes, including mean differences in MWT and ESS scores and relative risk values for adverse events, between the 150 and 300 mg dosages (77). Significantly, it also highlighted a rising discontinuation rate with increasing dosages. When compared to other wakefulness-promoting drugs such as modafinil, pitolisant, and sodium oxybate, solriamfetol showed superior improvements in both MWT and ESS scores. The adverse event relative risk was lower than modafinil and sodium oxybate but higher than pitolisant.

Research led by Vinckenbosch explored the effects of solriamfetol on the road-driving behavior of OSA patients. Utilizing the standard deviation of lateral position as the primary measure, Solriamfetol notably improved both driving performance and alertness compared to a placebo (78). Badran’s study also highlighted its positive influence on cognitive deficits and anxiety behaviors induced by chronic intermittent hypoxia in mouse models (79). Both short-term (12 weeks) and long-term (up to 52 weeks) trials showcased robust efficacy in ameliorating daytime activities and work efficiency in OSA patients with excessive daytime sleepiness, as assessed by MWT and ESS scores (80–83).

**FIGURE 1:**
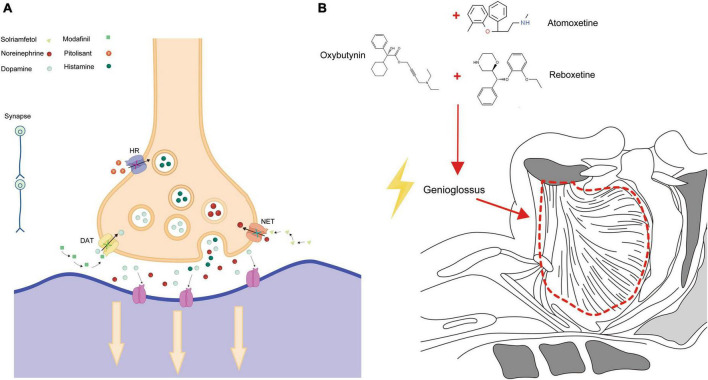
Pharmacological action in OSAS treatment. **(A)** Depicts the specific action mechanisms of Modafinil, Pitolisant, and Solriamfetol in treating OSAS. **(B)** Illustrates the broader pharmacological pathways and interactions of these medications.

Adverse reactions associated with solriamfetol most commonly included headaches, nausea, and anxiety. These reactions peaked in the first week post-administration and mostly resolved within a week. While the incidence of severe side effects was comparable to the placebo group, doses exceeding the recommended 150 mg posed an increased risk of side effects (84, 85). Modafinil, pitolisant, and solriamfetol are medications that play a key role in the treatment of OSAS. Their mechanism of action for managing OSAS is depicted in Figure 1A. These drugs work by targeting specific pathways and receptors involved in sleep regulation, promoting wakefulness, and reducing excessive daytime sleepiness.

### 4.4 Reboxetine combined with oxybutynin: a potential dual-therapy for OSAS

In the pathophysiology of OSAS, there is a simultaneous interplay between pharyngeal changes and alterations in the control of upper airway muscles during sleep. It is estimated that over 30% of patients experience a reduction in the activity of the upper airway dilator muscles as a result of the narrowing of the airway (86, 87). However, to date, no drug has been approved to treat OSAS by enhancing the activity of the upper airway muscle. Nevertheless, animal studies have demonstrated that enhancing genioglossus muscle activity during both wakefulness and sleep can alleviate symptoms of OSAS (88). Research indicates that both noradrenergic and antimuscarinic agents modulate the genioglossus muscle during both non-REM and REM sleep (89).

Reboxetine, a noradrenaline reuptake inhibitor, enhances the activity of the central neurotransmitter, offering promise as a new-generation antidepressant to improve patient mood. A study by Lim et al. (90) found that when combined with oxybutynin, an antimuscarinic agent, reboxetine can modulate genioglossus muscle activity during sleep, improve airway function, and alleviate OSAS symptoms (91). This improvement is attributed to the increase in loop gain and a reduction in the ventilatory response during wakefulness (90). This drug combination significantly improves nocturnal hypoxemia in severe OSAS patients and reduces the AHI index to <15 events/hour, with a remarkable 59% reduction in AHI for 81% of patients within a week. Although many trials have shown that standalone drug treatments aren’t significantly effective, this drug combination has demonstrated marked efficacy, reducing the severity of OSAS. However, this combined therapy is accompanied by certain adverse reactions, including insomnia, dry mouth, constipation, and excessive sweating. Currently, its mid-term effects and optimal dosages remain uncertain, necessitating further randomized controlled trials. These trials should encompass longer treatment durations, varying dosages, and larger sample sizes.

### 4.5 Combined therapy of atomoxetine and oxybutynin

Atomoxetine, a selective noradrenaline reuptake inhibitor, is predominantly employed in the clinical treatment of attention-deficit hyperactivity disorder (92). In OSAS patients, its therapeutic mechanism aligns with the above-mentioned rationale. Moreover, *in vitro* experiments have demonstrated that Atomoxetine can inhibit G-protein-coupled inwardly rectifying potassium channels. These channels play a pivotal role in reducing the excitability of hypoglossal motor neurons, thereby diminishing pharyngeal muscle tone during sleep and influencing sleep patterns (88, 93).

Oxybutynin, traditionally utilized for treating urinary disorders such as overactive bladder and urinary urgency, acts as an antimuscarinic agent. It exerts a potent muscle-relaxant effect on smooth muscles. Furthermore, antimuscarinics obstruct the inhibitory effect of acetylcholine on upper airway muscle tone during REM sleep (89).

Consequently, therapeutic interventions targeting the upper airway muscle group can effectively mitigate symptoms in patients with mild to moderate anatomical deficiencies (94–96). Montemurro found that concurrent evening administration of Atomoxetine and Oxybutynin could significantly reduce or even eliminate the severity of symptoms in patients. The fixed-dose combination, known as AD036 (comprising 80 mg Atomoxetine and 5 mg Oxybutynin), is still under development (96). However, preliminary trials, especially in patients with moderate OSAS, have indicated promising therapeutic outcomes. Another formulation, AD109 at higher doses (75/2.5 mg), has shown analogous efficacy (94, 97). Moreover, further studies have emphasized that the therapeutic potency of the Atomoxetine-Oxybutynin combination surpasses that of other antimuscarinic drugs by a considerable margin (98). As for side effects, patients may experience nausea, dry mouth, fatigue, and reduced appetite, but these typically subside within 2 weeks of medication commencement. No severe adverse reactions have been reported so far, possibly due to the short treatment duration.

At present, most studies on the Atomoxetine-Oxybutynin combination span just one night, with a relatively small sample size. Only one research endeavor has exceeded a month in duration (99). Given that OSAS is an irreversible chronic condition, it’s imperative to conduct more extensive evaluations to gauge long-term efficacy and safety (94). Oxybutynin, Atomoxetine, and Reboxetine are three medications that have been found to have an impact on OSAS. The action mechanisms by which these drugs exert their effects on OSAS are primarily depicted in Figure 1B. This figure serves as a visual representation of the intricate processes and pathways involved in how Oxybutynin, Atomoxetine, and Reboxetine interact with the condition, ultimately leading to potential therapeutic benefits for individuals suffering from OSAS.

### 4.6 Pharmacological advancements in OSAS treatment

Modafinil, armodafinil, pitolisant, and solriamfetol are currently authorized for managing a range of sleep disorders, as evidenced by their formal approval status. In contrast, reboxetine, oxybutynin, and atomoxetine are under investigation, with ongoing research and clinical trials assessing their effectiveness in the treatment of OSA, pending formal endorsement for this particular indication. Orexin neurons are primarily located in the hypothalamus and can be classified into two types: Type A and Type B. These neurons are associated with sleep-wake cycles, cardiopulmonary function, and autonomic regulation (100). Animal studies have demonstrated that the long-term effects of sleep fragmentation and intermittent hypoxia can lead to axonal lesions in orexin neurons, resulting in irreversible damage and subsequent impairment of the brain’s sleep-wake state (101, 102). Hence, targeting orexin in the brain holds significant potential for the development of novel therapeutic drugs.

Danavorexton is a selective agonist for the orexin-B receptor, which can help sustain and promote wakefulness. It has been shown to improve sleep quality in patients with narcolepsy and OSAS, reducing the residual EDS these patients experience (103–105). Another drug, Daridorexant, an antagonist for both orexin receptors, has been observed to improve the AHI and nighttime blood oxygen saturation in patients with mild to moderate conditions (106, 107).

Furthermore, medications like acetazolamide (108), non-benzodiazepines (109), and intranasal corticosteroids (110) have shown promise in clinical trials. While no significant adverse reactions were reported, their long-term efficacy and safety still require further investigation. A novel treatment approach involving the combined use of atomoxetine and fesoterodine has also shown an overall improvement in the severity of OSAS in patients (111). In summary, based on the pathological mechanisms of OSAS, the development and application of corresponding drugs are anticipated to become a primary choice for enhancing the quality of life of patients.

## 5 Conclusion

Based on the information presented, OSAS, as a chronic disease, affects multiple organ systems. In severe cases, it can lead to cognitive impairments and an increased risk of cardiovascular diseases (112, 113). As a supplement to CPAP therapy, pharmacological interventions have been employed to address the persistent excessive sleepiness in those diagnosed with OSAS (114). The use of modafinil and armodafinil has been shown to significantly improve symptoms of excessive daytime sleepiness, boost focus and vigilance, and improve overall clinical conditions as assessed by the CGI-C (Clinical Global Impressions–Improvement Scale). However, there has been no confirmed improvement in life quality or other cognitive areas such as memory and executive functions (115). CPAP is currently the primary treatment and effectively alleviates most of the patient’s symptoms, the long-term compliance associated with its use remains a challenge (116). Therefore, the development of new drugs, especially targeting pathological mechanisms such as noradrenaline and orexin in the brain, and upper airway muscle tone, is of paramount importance and may emerge as an alternative to CPAP in the future.

However, most drug trials are of short duration, which limits their primary therapeutic application. Key metrics, such as reducing the AHI value, improving EDS symptoms, and increasing oxygen levels, require long-term studies for OSAS severity. As our understanding of the disease mechanism deepens, future treatments might be tailored based on individual OSAS phenotypes. Whether it’s monotherapy or combined drug therapy, these advancements represent a step in a more promising direction for patients.

## Data availability statement

The original contributions presented in the study are included in the article/supplementary material, further inquiries can be directed to the corresponding authors.

## Author contributions

JL: Conceptualization, Formal Analysis, Writing – original draft. XY: Investigation, Resources, Writing – original draft. GL: Funding acquisition, Project administration, Writing – review and editing. PL: Data curation, Project administration, Supervision, Writing – review and editing.
